# An open-source controller to build a dynamic light intensity setup

**DOI:** 10.1186/s13007-024-01159-6

**Published:** 2024-02-28

**Authors:** Ludovico Caracciolo, John Philippi, Tom P. J. M. Theeuwen, Herbert van Amerongen, Jeremy Harbinson

**Affiliations:** 1 Laboratory of Biophysics, Wageningen University, 6700 ET Wageningen, The Netherlands; 2 MicroSpectroscopy Research Facility, Wageningen University, 6700 ET Wageningen, The Netherlands; 3 Jan IngenHousz Institute, Bornsesteeg 48, 6708 PE Wageningen, The Netherlands

**Keywords:** Fluctuating light, Photosynthesis, Light controller, Open-source

## Abstract

**Background:**

The development and physiology of plants are influenced by light intensity and its changes. Despite the significance of this phenomenon, there is a lack of understanding regarding the processes light regulates. This lack of understanding is partly due to the complexity of plant’s responses, but also due to the limited availability of light setups capable of producing specific light patterns.

**Results:**

While unraveling the complexities of plant responses will require further studies, this research proposes a simple method to implement dynamic light setups. In this study, we introduce two distinct electronic circuits that are cost-effective and enable the control of a dimmable power supply.

**Conclusion:**

This method enables the generation of intricate light patterns and rapid intensity fluctuations, providing a means to investigate how plants respond and develop when exposed to dynamic light conditions.

**Supplementary Information:**

The online version contains supplementary material available at 10.1186/s13007-024-01159-6.

## Background

Light intensity is an important parameter that shapes the physiology and development of plants [1–3] and an aspect of intensity are fluctuations in intensity. Plants respond to the fluctuation of light intensity through physiological changes ranging from short-term responses to longer-term adaptation. For example, the fluctuation of light intensity can induce changes in the chlorophyll *a*/*b* ratio, leaf thickness, and plant biomass [4–6]. To reproducibly study plant physiological processes, plants are often grown in controlled conditions. The light intensity in these controlled environment spaces is typically kept constant during the photoperiod. However, in nature irradiance can vary significantly within seconds, due to factors such as shading from other leaves or passing clouds, so account should be taken of plant responses to fluctuating irradiances (e.g. in 7). This difference between controlled environment systems and nature cause certain discrepancies between the observations made on plants grown in field and in controlled conditions [8]. To better understand how plants might operate in nature, it is desirable to mimic the natural dynamic light regime when growing plants under artificial irradiance. The effect of fluctuating irradiance on plant physiology, and most conspicuously on photosynthesis, has been recognized as a major knowledge gap in our understanding of plant/environment interactions as well as a path to crop yield improvement.

Many photosynthetic processes are affected by fluctuating irradiance. For example, when exposed to an increase in irradiance that results in carbon assimilation being no longer wholly light-limited, plants and other photosynthetic organisms can activate photoprotective mechanisms called non-photochemical quenching (NPQ). NPQ safely dissipates some of the excess excited states of chlorophyll formed in photosystem II (PSII) as heat. This dissipation is believed to lower photodamage in PSII [9]. Some components of NPQ relax when the light intensity decreases but this response can be relatively slow compared to the rate of decrease of irradiance. This results in PSII light-use efficiency becoming limiting for photosynthesis and, consequently, reduced CO_2_ fixation and biomass productivity. Knowledge of the underlying physiology and genes coding for proteins (including enzymes) connected with the formation and relaxation of NPQ allowed acceleration of the relaxation kinetics of NPQ via upregulation of a set of these genes. This resulted in a 15% increase in dry biomass of field-grown tobacco [10] and up to 33% increased yield of soybean seeds [11]. However, increasing the kinetics of NPQ relaxation may not always be the silver bullet to improve crop yield in the field; the same approach led to a decrease in biomass accumulation in *Arabidopsis thaliana* grown in fluctuating light conditions [12]. A deeper understanding of the effect of fluctuating light on photosynthetic performance is therefore needed to improve crop yield.

Field experiments will always be required to confirm the extent to which a potentially adaptive aspect of plant physiology has an impact under field conditions. However, our understanding of the underlying mechanisms of these adaptive features of plant function of plant biology can be accelerated by conducting initial trials where other potentially affecting parameters can be controlled (i.e. temperature, watering, competition). Therefore, there is an increased need to have a fluctuating irradiance in controlled environment spaces. The ease with which the light intensity can be controlled in indoor experiments has improved tremendously over the last 30 years. This improvement can be attributed first to the introduction of dimmable ballasts for fluorescent tubes and high-pressure sodium lamps, and then more recently to the introduction of dimmable LED lighting systems. However, there remain limitations in the options and extent of control of irradiance that can be routinely achieved in typical controlled environment rooms.

One approach is to switch the light on and off to vary between two different light intensities [13–15]. This approach is simple to implement and cost effective although it does not allow mimicking of the more complex fluctuations obtained in nature. Another solution is to use commercial systems that can change the light intensity according to a schedule set by the user. This allows the growth of plants under simulated natural daylight conditions, at least as far as the maximum irradiance and the emission spectrum of the system permits [2, 6, 16]. Nonetheless, this flexibility comes at a price that is not always affordable. A cheaper alternative is to design a dimmable LED power supply that can dynamically adjust the light intensity (e.g. in 16). However, many plant scientists do not have enough knowledge of electronics to implement an approach of this kind.

A workaround is to exploit the ability of some commercial lighting systems to be dimmed by an external signal and to home-build only the circuit required to produce this controlling signal. Dimmable power supplies are available for most LED or fluorescence tube fixtures [18], allowing a simple and easy implementation of a fluctuating irradiance system that can be retrofitted to an existing controlled environment space. We have developed two circuits that can be used with dimmable power supplies for LEDs or fluorescent lamps to produce a dynamic light system. The objective of this work is to provide individuals who are not always comfortable with electronics with the essential knowledge to build a simple light controller that can be connected to an existing dimmable lighting system. This is accomplished by offering comprehensive explanations, open-source code, and a practical example of how to build a small and low-cost programmable lightning setup. Moreover, this setup can adjust light intensity within milliseconds, which is substantially faster than the 20 s reported recently [19]. Please note that while every effort has been made to ensure the accuracy and safety of the instructions provided, users must exercise caution and take full responsibility for their actions and any potential risks involved in replicating this circuit.

## Methods

The proposed dynamic light system is composed of a dimmable power supply, a light source, and a controller circuit. The type of power supply needed depends on the light source that needs to be driven (fluorescence, LED, etc.). Dimmable power supplies exist for a whole variety of lamps. In this work, we implemented a dynamic lighting system based on LEDs because of their wide availability and decreasing cost. Nonetheless, the same approach can also be used with other types of light sources if their power supply is dimmable.

The wide-scale use of LEDs as a light source in controlled environments for plant research is relatively new. To avoid confusion over terminology and the way LEDs function, a short overview on how LEDs work is provided hereafter. As light sources, LEDs need to be powered using direct current (DC) applied using the correct polarity. The power supply therefore converts alternating current (AC) supplied from the mains socket to DC. Within the operating limits of the device, the brightness or radiation output of an LED is almost linearly correlated to the current flowing through it. LEDs can be dimmed in two ways; first by controlling the current flowing through the LED or, second, by driving them with a current pulsed at a fixed frequency (usually>3kHz) but with a variable duty-cycle (i.e. the ratio between the on and off time of the pulses). The first method is referred to as Constant Current Reduction (CCR) while the latter is generally referred to as Pulse Width Modulation (PWM). The frequency of the PWM modulation is usually well above that for the human perception of flicker (which is about 25 Hz) and the perceived brightness depends on the duty-cycle. While PWM is often used in illumination engineering it is not suited for plant growth; some studies indicate that using PWM to control irradiance can induce physiological responses [20]. Therefore, the use of CCR is preferred to control irradiance in plant research unless the aim is to provide irradiance in the form of repetitive pulses. It is worth noting that the power supplies dimmable using CCR might use PWM as a control signal (i.e. the amplitude of the constant current is adjusted through a PWM control signal). In the method presented here, we will use a PWM only as a control signal so dimming of irradiance will always be achieved by reducing the current provided to the LEDs.

### Overview of the setup

**Fig. 1:**
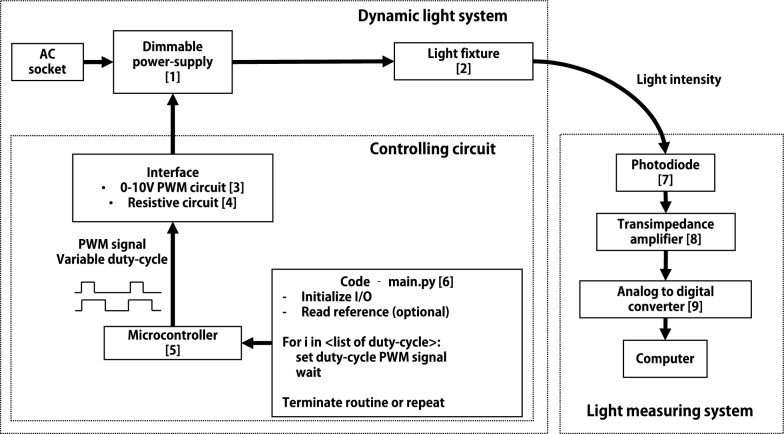
Overview of the dynamic light setup and light measuring system. LCM-60, Mean Well^®^ [1]; L2C5-30HG1203E0900, Lumiled^®^ [2]; the interfaces [3, 4] are reported in Figs. 2 and 4; RP2040, Raspberry Pi Foundation [5]; example code in Additional file 1 [6]; LI-COR^®^ quantum sensor [7]; DLPCA-200, Femto [8]; PICOLOG 1216, Pico Technology [9]

**Fig. 2:**
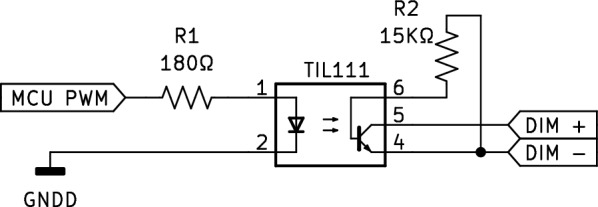
Circuit diagram of the 0–10 V PWM circuit. R_x_, resistors; TILL111, optocoupler

**Fig. 3:**
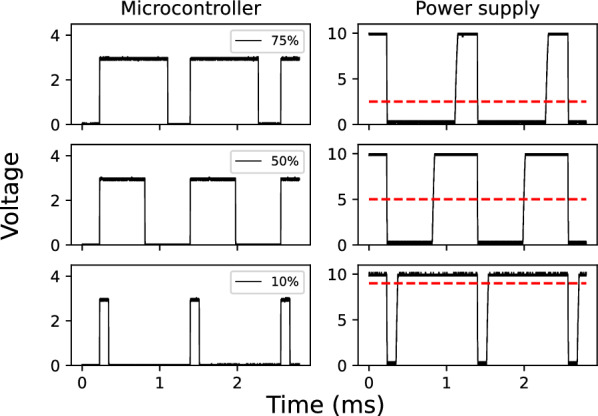
Oscilloscope measurement of the PWM signal on the microcontroller and power supply side. The dashed line indicates the average voltage sensed on the power supply control pins

**Fig. 4:**
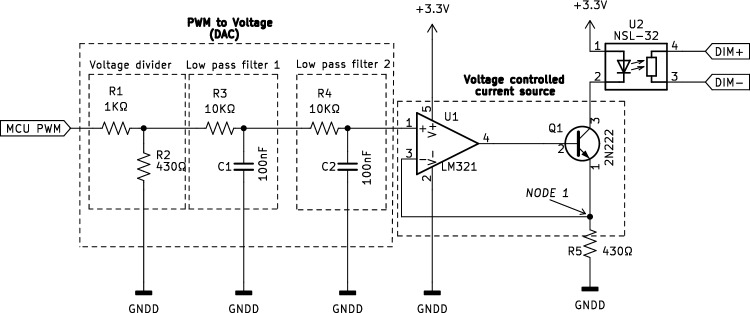
Circuit diagram of the resistive circuit. R_x_, resistors; C_x_, capacitors; U1, opamp; U2, optocoupled LDR; Q1, transistor

**Fig. 5:**
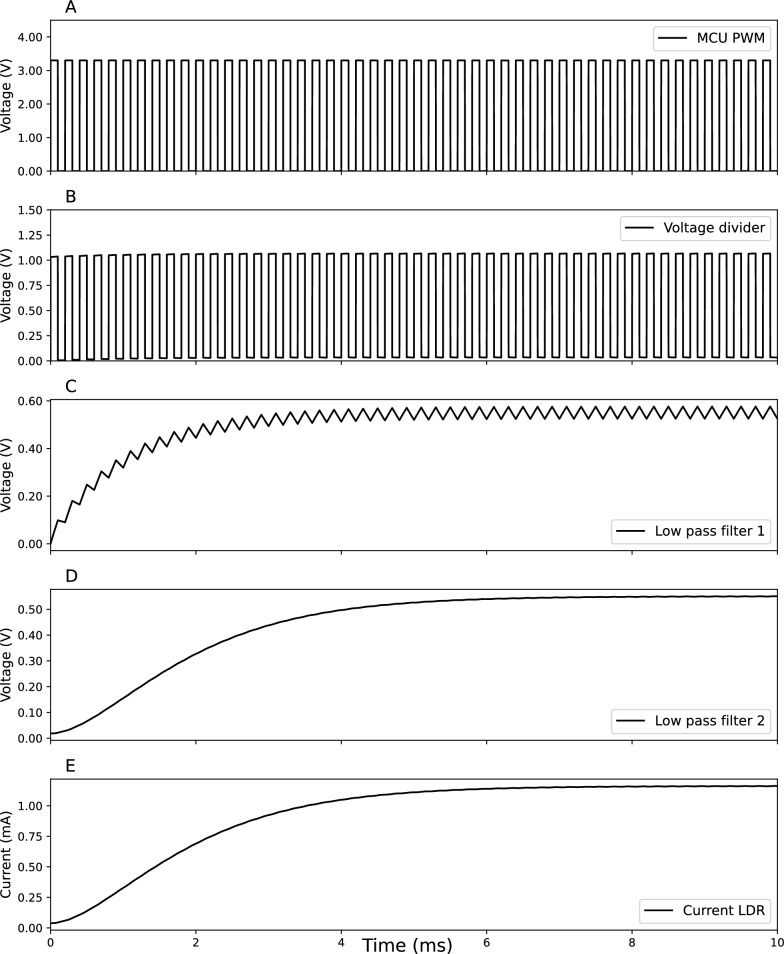
Difference in voltage/current in the different parts of the resistive circuit. At the output of the MCU (**a**). After the voltage divider (**b**). After the first LP filter (**c**). After the second LP filter (**d**). The current flowing on NODE 1 (**e**)

**Fig. 6:**
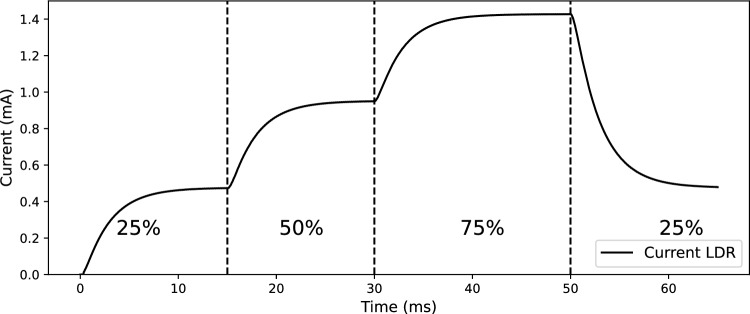
Current flowing through NODE 1 after a change of the PWM duty-cycle (in percentage)

Fig. 1 summarizes the dynamic light setup presented in this work. Commercial dimmable power suppliers have their dimming percentage usually controlled through the so-called 0–10V protocol. These power supplies have two control pins from which a 10V voltage is sourced. There are two commonly used ways of controlling the dimming percentage of a 0–10V dimmable power supply. One is to modulate the 10V on the power supply side in a PWM fashion way, the second is by changing an external resistance between the controlling pins [21]. Figs. 2 and 4 show, respectively, the interfacing circuits for a 0–10V PWM control and a resistive control. In both cases, the controlling signal is generated by a microcontroller (MCU) and is PWM modulated at a constant frequency (in the kHz range) with a variable duty-cycle. The percentage of the duty-cycle controls the current dimming of the power supply. In our case, an RP2040 (Raspberry Pi Foundation) was chosen as the MCU because of its low price and its use of Micropython, which is a dialect of Python designed for microcontrollers. Python is an easy to use and widely taught programming language. Any other MCU, however, that is able to generate a PWM output could have been used (e.g. an Arduino). To achieve dynamic fluctuations, the microcontroller follows an algorithm outlined in the main application code uploaded to the MCU (Fig. 1–[6], “main.py”). This program typically involves reading a value from an array or text file, changing the PWM duty-cycle based on the read value, and waiting for a specified duration before repeating the process. For complex fluctuations with thousands of different dimming percentages (i.e. while mimicking a natural light profile), the duty-cycle's values can be saved on an external drive (e.g. an SSD or an SD card). Both interface circuits use optically coupled components to electrically isolate the MCU from the power supply to allow communication between different voltage levels and to increase the safety of the system by largely isolating the low voltage home-built circuit from the mains voltage dimmable power supply (i.e 3.3 V and 10 V).

### Interface circuits

#### 0–10 V PWM interface

Fig. 2 shows the circuit for the 0-10V PWM interface (see Fig. 1–[3]). It is composed of two resistors and one optocoupled transistor (TIL111, ON Semiconductor/Fairchild). The dimming percentage of the power supply linearly depends on the voltage sensed between its control pin, averaged over time. The control pins of the power supply (DIM+, DIM−) are connected to the transistor side of the optocoupler. The base of the transistor (Fig. 2, TIL111 pin 6) is connected through a pull-down resistor to the emitter (Fig. 2, TIL111 pin 4), to decrease the fall time of the modulated pulse. The value of R1 was chosen according to the datasheet of the TIL111 to produce a 10mA current running through the optocoupler. Fig. 3 shows the voltage measured on the microcontroller side (Fig. 2, TIL111 pins 1 and 2) and on the power supply controlling pin side (Fig. 2, TIL111 pins 4 and 5).

#### Resistive interface

Fig. 4 shows the circuit for a resistive interface (see Fig. 1–[4]). The circuit is composed of five resistors, two capacitors, one operational amplifier (LM321, Texas Instrument), one NPN transistor (2N222, ON Semiconductor), and one optocoupled light-dependent resistor (NSL-32-R2, Advanced Photonix). While the 0–10V PWM interface is easy to implement, some dimmable power supplies might have an unstable output when controlled by the 0–10V PWM interface. The resistive interface controls the dimmable power supply output by changing the resistance between the power supply’s control pins. As resistance increases, the dimming percentage decreases. An optocoupled light-dependent resistor (optocoupled-LDR) was chosen as variable resistance. An LDR is a component that changes its resistance in response to the light intensity. This component has an LED and an LDR enclosed in a single package. When the LED is powered, the light intensity logarithmically decreases the resistance of the LDR. A Voltage Controlled Current Source (VCCS) is used in the circuit to drive at a constant current the LED in the optocoupled LDR. The VCCS is composed of an operational amplifier (U2), a transistor (Q1), and a resistance (R5). See the appendix for an explanation on how the VCCS works. A Digital to Analog Converter (DAC) is needed to provide a digitally controlled variable voltage. Since the RP2040 does not have an onboard DAC, we converted the high-resolution PWM (16bit) to a constant voltage by using two low-pass filters. Simulation made in LTspice^®^ (Analog Device) shows the operation of the circuits (Figs. 5 and 6). To avoid saturating the operational amplifier, the 3.3V amplitude of the PWM (Figure 5a), is reduced to 1 V through a voltage divider (Fig. 5b). The two first-order low-pass filters put in series smooth the square waves to a close-to stable voltage (Fig. 5c, d). In this way, we can generate an adjustable current flowing through the LDR that depends on the duty-cycle of the PWM (Fig. 6).

### Coding the MCU

In this work, the MCU uses a MicroPython program as firmware. When a MicroPython device starts up, it automatically looks for a file named “main.py” on its file system and executes the code within that file. Developers often write their main application logic in the “main.py” file, including initializing hardware components, configuring settings, and implementing the desired functionality. The Thonny IDE [22] was used to upload to the RP2040 the main application and, in some cases, the desired dimming values were stored in an additional text file saved in the MCU's memory. The MCU is programmed to initialize the I/O and generate a PWM signal at a fixed frequency. The duty cycle of PWM is adjusted based on predefined values, each of which is followed by a specific time delay before the process is repeated (for an example, see Additional file 1: Code 1). Due to the limited number of functions and libraries available by default in MicroPython, more complex fluctuations, such as a sinusoidally modulated irradiance, are best obtained by calculating the dimming steps on a computer and then providing them as a text file to MCU (see Additional file 1: Code 2 and 3). When generating a complex light profile with thousands of points (i.e. when mimicking diurnal light intensity), it is also easier to upload them to the MCU as an additional text file and then read them sequentially (see Additional file 1: Code 4). If the external text file is too large to fit the onboard memory of the MCU, an external memory device can be used (i.e. an SD card). Examples demonstrating SD card setup and reading in MicroPython for RP2040 microcontroller can easily be found online.

### Calibrating the setup

To achieve specific light intensities, often required in photosynthetic photon flux density (PPFD), it is necessary to calibrate the control circuits. The approach to calibration depends on the type of interface used and involves utilizing a photosynthetic active radiation (PAR) sensor, (e.g. Li-250, LI-COR Inc). In the case of the 0–10V PWM interface (Fig. 2), the duty cycle generated by the MCU is inversely equal to the dimming percentage of the power supply; for example, if the duty-cycle of the PWM generated by the MCU is 25%, the dimming percentage of the power supply will be 75%. In the case of the resistive interface (Fig. 4), a calibration table is required. A calibration table is made by sequentially changing the duty-cycle of the PWM generated by the MCU and noting the corresponding PPFD produced by the power supply. The relation between duty-cycle and dimming percentage is non-linear due to the characteristic of the optocoupled LDR (Fig. 4-U2).

### Light setup

The light control unit was used to control one high-power and one low-power light setup, respectively 3800 W and 25 W power output (Fig. 1–[1]). The high-power setup was composed of 6 Vypr V2 modules (Fluence), used in controlled environment rooms and greenhouses as light source. The low-power setup was comprised of a single LED (L2C5-30HG1203E0900, Lumiled) powered by a dimmable power supply (LCM40, Mean Well), which was used as an actinic light source in laboratory experiments.

### Light measurement

**Fig. 7:**
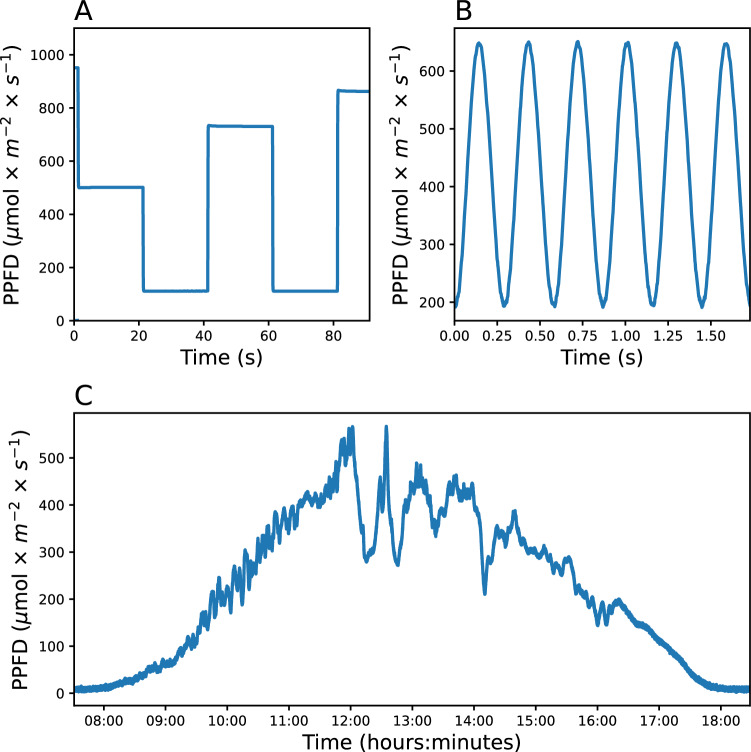
Different light patterns generated with the 0–10 V PWM interface controlling a benchtop light setup. Step-wise change in irradiance (**A**); sinusoidal fluctuation (**B**); mimicked natural light fluctuation (**C**)

The irradiance profiles generated by the controlling circuit were measured using a light-measuring setup made of off-the-shelf components (Fig. 1—[7–9]). A Li-Cor^®^ quantum sensor was connected to a commercial transimpedance amplifier (DLPCA-200, Femto), which has an output socket and also has a faster frequency response than the Li-Cor PAR meter (Li-250, LI-COR, Inc). The output was recorded using a datalogger with a sampling frequency of 1 kHz (PICOLOG 1216-Pico Technology). The Li-Cor^®^ sensor/Femto pair were calibrated using the same sensor with a Li-Cor readout meter (Li-250, LI-COR, Inc). The same off-the-shelf photodiode, transimpedance amplifier, and datalogger were used to measure a profile of natural irradiance from a north-facing window (coordinates 51.984620, 5.661515) with a sampling frequency of ~1, 5 Hz (see Additional file 2).

## Results

### Generating different patterns of light

The presented method was used to generate 3 different light patterns (Fig. 7) using a benchtop light setup composed of a white light LED and a 60W dimmable power supply. The power supply accepted a 0-10V PWM dimming protocol and was therefore controlled using the circuit of Fig. 2.

The first light pattern was a series of step-wise irradiance changes (Fig. 7A). The light setup was controlled to achieve 6 different intensities, respectively 100, 50, 10, 75, 10, and 90% of its maximum output. The MCU was programmed to change the duty-cycle of the PWM control signal 6 times with a delay of 20 s between each change (see Additional file 1: Code 1). A step-wise change of light intensity of this kind can be used in a growing environment to produce a steady light intensity or to expose plants to a sudden change of irradiance to assess the kinetics of a physiological response (e.g. changes in carbon assimilation rates, changes in quantum efficiency of photosystem II, etc.).

The second light pattern generated was a 4Hz sinusoidal fluctuation (Fig. 7B). When used as an actinic light pattern it can be used to study the frequency response of plant's photosynthesis through chlorophyll fluorescence or photoacoustic signal [23, 24]. For this more complex pattern, the microcontroller was programmed (see Additional file 1: Code 3) to iterate repetitively through an array of values encoding duty-cycles changing as one period of a sinusoid. The array of values encoding the duty-cycle was first generated on a PC using a Python script (see Additional file 1: Code 2). The frequency of the sinusoidal output depends on the delays coded between each change of duty-cycle. It is worth noting that while the circuit supports change of duty-cycles in less than milliseconds, the maximum achievable light fluctuation frequency mostly depends on the response time of the power supply (see controlling light setup section).

The last light pattern mimicked a natural irradiance recorded during a day (Fig. 7C). This light pattern has therefore a profile of the kind to which a plant could be exposed in nature. Because of the high number of values in the light intensity dataset (above 43000 points), the dataset was supplied to the microcontroller memory as an additional text file along with the main program (see Additional file 1: Code 4).

### Controlling different light setup

The flexibility of the system was assessed by controlling two light setups with different power outputs; a low-power setup (30 W) used as a laboratory light source, and a high-power setup (3600 W) used in a growth chamber. The low-power light setup was the one used to generate the light pattern in Fig. 7. The high-power light setup consisted of six LED arrays powered by six power supplies (Vypr V2 modules, Fluence). We tested both light setups using the 0-10V PWM circuit (Fig. 2) programmed to generate a light pattern with sequential step-wise fluctuation (see Additional file 1: Code 1).

**Fig. 8:**
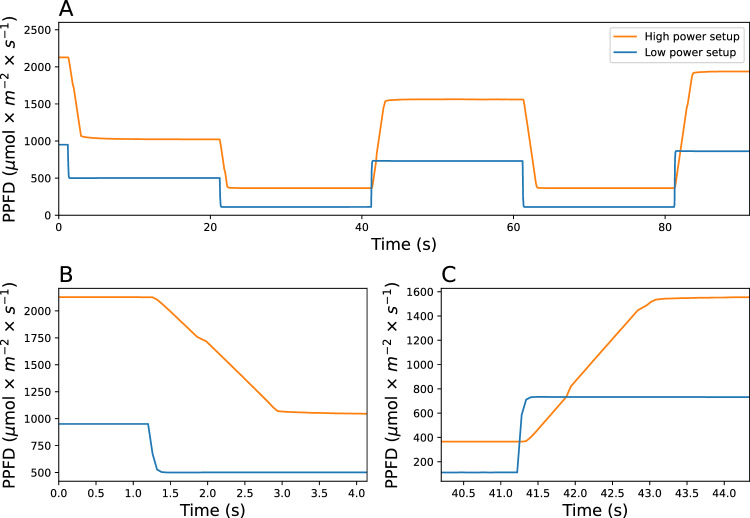
Step-wise pattern generated with a high power and low power light setup controlled by the 0–10 V PWM interface (**a**). Close-up view of the descent time during the transition from 100 to 50% dimming percentage (**b**). Close-up view of the ascent time during the transition from 10 to 75% dimming percentage (**c**)

There is a noticeable difference in the time response of the irradiance changes between the high-power and low-power configurations (Fig. 8), although the control circuit used was the same for both. A longer time response limits the speed at which a change in irradiance of a certain amplitude can be achieved. The response time for changes to the irradiance output of the high-power setup was longer when compared to the low-power setup. The longer response time depends on the electrical design of the power supply rather than the controller. Some power supplies, especially those made to drive large loads in electrically noisy environments, are designed to prevent rapid or sudden fluctuations in the output current by damping the response of the power supply [25]. Note, that power supply with a longer time response still offers the capability of achieving rapid fluctuations, albeit with a smaller amplitude.

## Discussion

In plant research, understanding and controlling light intensity is crucial for gaining insights into plant growth, development, and responses to environmental cues. However, there is a gap between the limited commercial options available for controlling light intensity and the actual requirements of plant research. To bridge this gap, in the last years, several inexpensive laboratory-built dynamic lighting systems have been developed. While these systems offer affordable solutions, they often prioritize simplicity over the ability to create a complex irradiance profile. For example, some systems were designed to produce step changes between two limiting (maximum and minimum) irradiances [14]. In contrast, our method enables an almost continuous dimming of the light (with a 16-bit resolution in our current design), opening the possibility of generating a wealth of different light profiles. Other approaches directly switch the current flowing through the LED on and off using a PWM signal, resulting in a dynamic but pulsed control of the light intensity [26]. Employing such pulsed control for growing or studying plants is problematic as it may impact the physiological processes of the plants [20]. While our method also uses PWM, it is only used as control feedback for the power supply and the current to the LED is kept constant. Note, however, that some power supplies may flicker at a dimming percentage below 10% due to instability of the regulation of the power supply.

In nature, most light flecks take place in less than 2 s, but there is a lack of studies on how plants respond to fluctuations shorter than 20 s [19]. Therefore, achieving rapid fluctuations was a requirement in the design of our method. The 0–10 V PWM circuit (Fig. 2) and the resistive circuit (Fig. 4) exhibit different response times when changing the controlling signal. The 0-10V PWM circuit can, in principle, switch between two control signals within microseconds, while the resistive circuit requires approximately 10–15 ms to stabilize to a new signal (Fig. 6). However, the main limitation to achieving fast fluctuations depends on the electrical design of the power supply (Fig. 8); some power supplies are designed to damp changes of irradiance to avoid fluctuations that could be undesirable in certain environments as offices or domestic lightning. On the other hand, even with these power-supply design limitations, the fluctuations below 1 s are easily achievable with the method we use. An additional strength of the method we have developed lies in its ability to control a wide range of power supplies including those with very different wattages. The electrical isolation between the control circuit and the power supply enables its integration into light setups found in greenhouses or climate chambers. The primary limitation in the implementation of our method is the requirement for a dimmable power supply. Fortunately, the majority of existing light setups can be externally dimmed.

The systems we have developed can be particularly useful in photosynthesis research, where there is a growing interest in understanding the effects of fluctuating light on photosynthesis. To date the method described in this study has been used in three distinct research projects in which changes in growth light pattern were shown to affect photosynthesis and plant development [27–29]. In the first investigation, differing profiles of irradiance, each providing the same daily integral of irradiance, were compared. Three different light growth conditions, one sinusoidal, one square wave, and one fluctuating were tested on a panel of different *Arabidopsis thaliana* genotypes. The sinusoidal light regime was shown to improve shoot biomass in comparison to square wave light regimes [27]. In the other two projects, the effect of specific allelic variation in different environmental conditions was assessed using *Arabidopsis thaliana* genotypes grown in a range of constant and fluctuating light conditions. One light regime included large fluctuations every 100ms, which resulted in significant biomass differences in plants with allelic variation for an NDH subunit. This finding was essential to reveal the possible relevance of the NDH complex in field conditions [28]. Altogether, this shows how our method can be useful to reveal novel physiological insights as well as reveal relevant genetic variants that can be used for crop improvements. Finally, our method could be used in modulating the irradiance in systems to measure the frequency-dependent photosynthetic responses (Fig. 7).

## Conclusion

In conclusion, our work provides an outline of how to build a fluctuating light setup along with the open-source code needed for its control. We expect that this will simplify the implementation of a dynamic light system for researchers. Programmable light setups that generate reproducible and complex patterns of light intensity are necessary for experiments aimed at disentangling the effects of the multiple mechanisms affecting the rates of carbon assimilation. However, light intensity is not the only variable parameter to affect plant physiology in nature. Fluctuations in CO_2_ mole fraction or, water vapor mole fraction between a leaf and the surrounding air, or fluctuations in temperature or light spectrum are all factors that also affect carbon assimilation [30]. To fully understand plant's physiological response to fluctuations in natural settings more studies are required in controlled environments with realistic dynamic environmental parameters.

### Supplementary Information

**Additional file 1.** Code examples for the microcontroller.

**Additional file 2.** Normalized fluctuating light dataset.

## Data Availability

All data generated or analyzed during this study are included in this published article and its Additional file 2 or from the corresponding author on reasonable request.

## Abbreviations

LED: Light Emitting Diode
NPQ: Non-photochemical quenching
DC: Direct current
AC: Alternated current
PWM: Pulse width modulation
CCR: Constant current regulation
MCU: Microcontroller unit
LDR: Light dependent resistor
VCCS: Voltage controlled current source
I/O: Input/output
PPFD: Photosynthetic photon flux
