# Supplementary material for: An open-source controller to build a dynamic light intensity setup
Source: Plant Methods. 2024 Feb 28;20:35. doi: 10.1186/s13007-024-01159-6 (PMC10902962; doi:10.1186/s13007-024-01159-6)
Supplement: Supplementary file 1 — Additional file 1. Code examples for the microcontroller. [file 13007_2024_1159_MOESM1_ESM.docx]

**Appendix**

Way of working of VCCS

The operational amplifier in the VCCS endeavors to keep the same voltage between its inverting pin (the ‘-‘ pin, number 3, Fig.4) and its non-inverting pin (the ‘+’ pin, number 1, Fig.4). If 800mV is applied to the non-inverting input, the operational amplifier (U2) will adjust the current flowing through Q1 so that the inverting input will reach 800mV. This also means that on node 1 (Fig. 4) there will be 800mV. The current flowing through R5, and therefore through U2 will be:

I_OptoLDR=V_Node1/R_R5 =800mV/430Ω≈2mA

According the NSL-32-SR2 datasheet, when the LED’s side is driven with 2mA, the LDR resistance will be roughly 80Ω.

from machine import Pin # import library required for the script

import time

pin15 = machine.Pin(15, machine.Pin.OUT) # initialize pin 15 as output

pin15 = machine.PWM(pin15) # set the initialized pin as PWM output

pin15.freq(1000) # set the frequency of the PWM signal to 1KHz

delay = 20 # time delay

values = [0, 32768, 58982, 16384, 58982, 6554] # duty-cycle values

for value in values:

pin15.duty_u16(value) # set the PWM duty-cycle

time.sleep(delay) # sleep for 20 seconds

Code 1 – MCU micropython code to generate step-wise switch light pattern

Code 1 exemplify the steps followed by the MCU to create a 6 steps light fluctuation. The I/O on pin15 is first initialized and set to generate a PWM signal at a 1kHz frequency. The MCU iterates through 6 values, set the PWM’s duty-cycle to that value through the command gpio.duty_u16() and wait for a predefined amount of time. The variable value is a number that sets the duty-cycle, which ranges from 0 to 65535 (unsigned 16bit). The values 0 and 65535 stands for a duty-cycle of 0% and 100% respectively. As a practical example, to encode a duty-cycle of 75% the value should be set to 49151 (65535 * 0.75 = 49151, rounded to the closest integer).

import numpy as np

from math import pi, sin

pwm_max = 65535 # desired maximum PWM duty-cycle value

pwm_min = 32768 # desired minimum PWM duty-cycle value

x = np.arange(0, 2*pi, 0.1) # generate numbers ranging between 0 and 2ϖ with a 0.1 steps

y = [sin(i) for i in x] # generate one values for sine period

y = (y - np.min(y)) / (np.max(y) - np.min(y)) # normalize y between 0 and 1

y *= pwm_max - pwm_min # normalize the values of the sine wave in the duty-cycle range

y += pwm_min

Code 2 –Python code to generate values of duty-cycle encoding for a sine-wave shaped light profile.

from machine import Pin

import time

pin15 = machine.Pin(15, machine.Pin.OUT)

pin15 = machine.PWM(pin15)

pin15.freq(1000)

y = [x1,x2,x3, ect..] # array previously generated using code 2

delay = 0.050

while True:

for value in y:

pin15.duty_u16(value) # set the duty-cycle

time.sleep(delay) # sleep

Code 3 – MCU micropython code use the array generated in code 2 to produce periodic sinewave-shaped light profile.

from machine import Pin

import time

pin15 = machine.Pin(15, machine.Pin.OUT)

pwm15 = machine.PWM(pin15)

pwm15.freq(1000)

delay = 1

with open("reference.txt" ,"r") as f:

for value in f:

value = int(value) # convert the read value to integer

pwm15.duty_u16(value) # set the duty-cycle

time.sleep(delay) # sleep

Code 4 – MCU micropython code to generate a sinusoid light pattern
